# Deep Learning for Automatic Segmentation of Oral and Oropharyngeal Cancer Using Narrow Band Imaging: Preliminary Experience in a Clinical Perspective

**DOI:** 10.3389/fonc.2021.626602

**Published:** 2021-03-24

**Authors:** Alberto Paderno, Cesare Piazza, Francesca Del Bon, Davide Lancini, Stefano Tanagli, Alberto Deganello, Giorgio Peretti, Elena De Momi, Ilaria Patrini, Michela Ruperti, Leonardo S. Mattos, Sara Moccia

**Affiliations:** ^1^ Department of Otorhinolaryngology—Head and Neck Surgery, ASST—Spedali Civili of Brescia, University of Brescia, Brescia, Italy; ^2^ Department of Otorhinolaryngology—Head and Neck Surgery, IRCCS San Martino Hospital, University of Genoa, Genoa, Italy; ^3^ Department of Electronics, Information and Bioengineering, Politecnico di Milano, Milan, Italy; ^4^ Department of Advanced Robotics, Istituto Italiano di Tecnologia, Genoa, Italy; ^5^ The BioRobotics Institute, Scuola Superiore Sant’Anna, Pisa, Italy; ^6^ Department of Excellence in Robotics and AI, Scuola Superiore Sant’Anna, Pisa, Italy

**Keywords:** oral cancer, oropharyngeal cancer, segmentation, machine learning, neural network, deep learning, narrow band imaging

## Abstract

**Introduction:**

Fully convoluted neural networks (FCNN) applied to video-analysis are of particular interest in the field of head and neck oncology, given that endoscopic examination is a crucial step in diagnosis, staging, and follow-up of patients affected by upper aero-digestive tract cancers. The aim of this study was to test FCNN-based methods for semantic segmentation of squamous cell carcinoma (SCC) of the oral cavity (OC) and oropharynx (OP).

**Materials and Methods:**

Two datasets were retrieved from the institutional registry of a tertiary academic hospital analyzing 34 and 45 NBI endoscopic videos of OC and OP lesions, respectively. The dataset referring to the OC was composed of 110 frames, while 116 frames composed the OP dataset. Three FCNNs (U-Net, U-Net 3, and ResNet) were investigated to segment the neoplastic images. FCNNs performance was evaluated for each tested network and compared to the gold standard, represented by the manual annotation performed by expert clinicians.

**Results:**

For FCNN-based segmentation of the OC dataset, the best results in terms of Dice Similarity Coefficient (Dsc) were achieved by ResNet with 5(×2) blocks and 16 filters, with a median value of 0.6559. In FCNN-based segmentation for the OP dataset, the best results in terms of Dsc were achieved by ResNet with 4(×2) blocks and 16 filters, with a median value of 0.7603. All tested FCNNs presented very high values of variance, leading to very low values of minima for all metrics evaluated.

**Conclusions:**

FCNNs have promising potential in the analysis and segmentation of OC and OP video-endoscopic images. All tested FCNN architectures demonstrated satisfying outcomes in terms of diagnostic accuracy. The inference time of the processing networks were particularly short, ranging between 14 and 115 ms, thus showing the possibility for real-time application.

## Introduction

Surgical data science (SDS) (1) is an emerging field of medicine aimed at extracting knowledge from medical data and providing objective measures to assist in diagnosis, clinical decision making, and prediction of treatment outcomes. In this context, image segmentation, an essential step in computer vision, can be defined as the task of partitioning an image into several non-intersecting coherent parts (2). It is also well known that segmentation is a prerequisite for autonomous diagnosis, as well as for various computer- and robot-aided interventions. Many methodologies have been proposed for image segmentation (3), but the most recent and successful approaches are based on fully convolutional neural networks (FCNNs), applying convolutional filters that learn hierarchical features from data (i.e., input images), and then collecting them in maps. In general, a high number of filters will give better results up to a certain point, when a further increase in their number either does not improve the segmentation performance, or deteriorates it (4).

FCNNs applied to video-analysis are of particular interest in the field of head and neck oncology, since endoscopic examination (and its storage in different ways and media) has always represented a crucial step in diagnosis, staging, and follow-up of patients affected by upper aero-digestive tract cancers. In this view, Narrow Band Imaging (NBI) represents an already consolidated improvement over conventional white light endoscopy, allowing for better and earlier identification of dysplastic/neoplastic mucosal alterations (5–8). However, so far, NBI-based endoscopy remains a highly operator-dependent procedure, and its standardization remains particularly challenging, even when employing simplified pattern classification schemes (9–14). In fact, even though such bioendoscopic tools are aimed at identifying pathognomonic superficial vascular changes in forms of abnormal intrapapillary capillary loops, a relatively long learning curve and intrinsic subjectivity of subtle visual evaluations still hamper their general and widespread adoption in daily clinical practice. Furthermore, subtle differences in NBI patterns according to the head and neck subsite to be analyzed have also been described. This is especially true considering the oral cavity (OC) in comparison with other upper aero-digestive tract sites (9).

The aim of this study was to test FCNN-based methods for semantic segmentation of early squamous cell carcinoma (SCC) in video-endoscopic images belonging to OC and oropharyngeal (OP) subsites to pave the way towards development of intelligent systems for automatic NBI video-endoscopic evaluations.

## Materials and Methods

**Figure 1:**
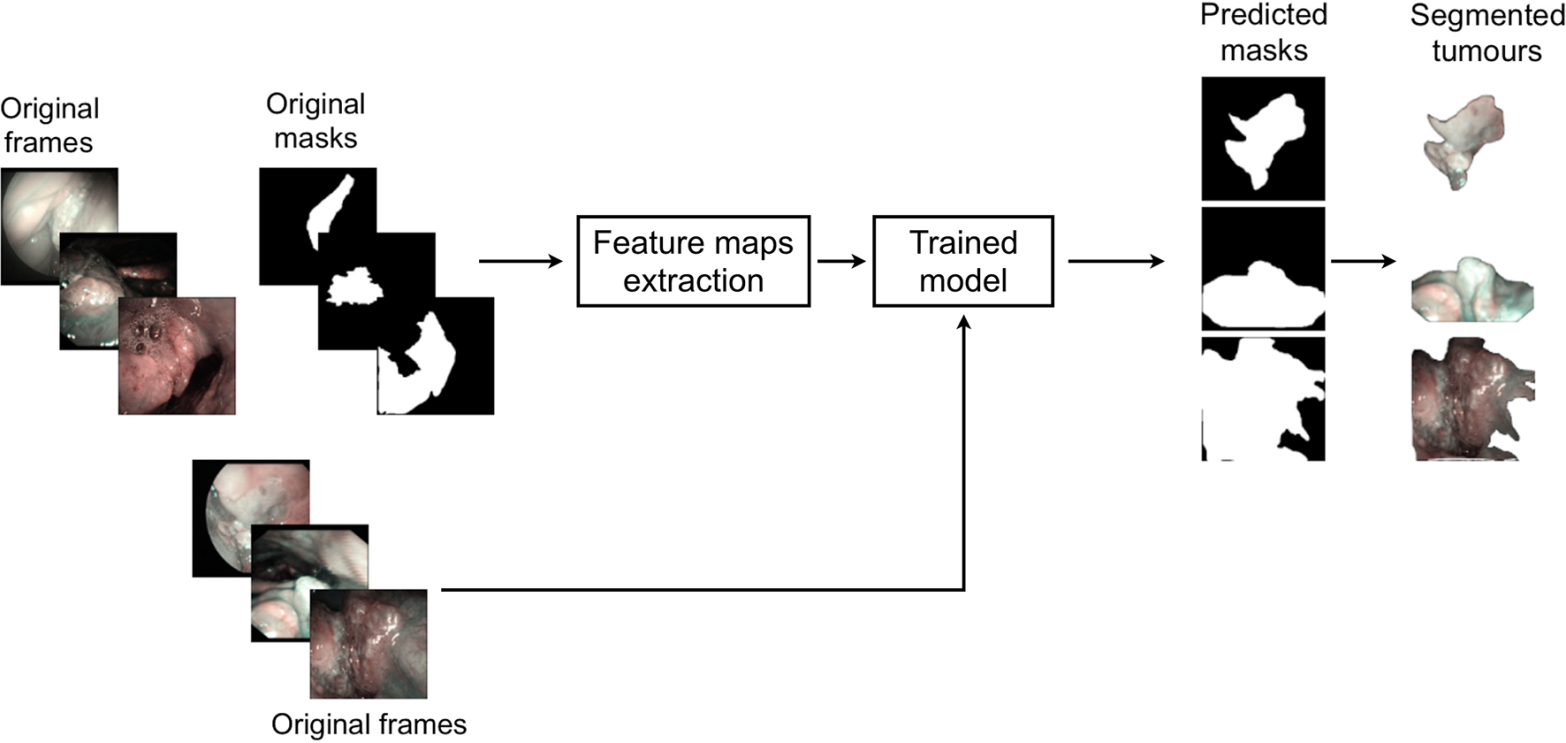
Workflow of the approach used for detection of mucosal SCC in videoendoscopic frames by NBI.

This study was performed following the principles of the Declaration of Helsinki and approved by the Institutional Review Board, Ethics Committee of our academic hospital (Spedali Civili of Brescia, University of Brescia, Brescia, Italy). The workflow of the approach used is shown in **Figure 1**. In particular, informative NBI frames were selected from videos of OC and OP SCC through a case by case evaluation (“Original frames”, **Figure 1**). Each frame was manually annotated by an expert clinician contouring the lesion margins, thus creating a mask referring to every frame (“Original mask”, **Figure 1**). The original frames and masks were then employed to train the FCNNs in order to obtain an automatic tumor segmentation.

### Mucosal Cancer Segmentation

Two datasets were retrieved from the institutional registry analyzing 34 and 45 NBI endoscopic videos of OC and OP, respectively. Each video was from a different patient affected by SCC, clinically presenting as a leuko- or erythroplastic lesion. Image acquisition was performed at the Department of Otorhinolaryngology – Head and Neck Surgery, ASST Spedali Civili, University of Brescia, Brescia, Italy between January 2010 and December 2018. Only video-endoscopies of biopsy-proven OC and OP SCC were included in the study. Patients with previous surgical and/or non-surgical treatments for tumors of these anatomical sites and frankly ulcerated neoplasms with significant loss of substance were excluded from the analysis.

All videos were acquired under white light and NBI by a rigid telescope coupled to an Evis Exera II HDTV camera connected to an Evis Exera II CLV-180B light source (Olympus Medical Systems Corporation, Tokyo, Japan). From the total amount of frames constituting the NBI videos, non-informative frames (i.e., blurred, out of focus, dark, or with signs of bleeding) were discarded through a case by case evaluation. After this selection process, the dataset referring to the OC was composed of 110 frames, while a total of 116 frames composed the OP dataset. **Table 1** shows the number of frames tested per patient for each dataset and the relative total amount of frames and patients involved. Each frame in these databases was manually annotated by an expert clinician contouring the lesion margins. The correspondent mean lesion size in percentage of pixels with respect to the entire frame size for each dataset is reported in **Table 2**.

**Table 1:** Investigated datasets for mucosal SCC segmentation task and corresponding number of NBI videoframes per patient.

Oral cavity			
	No. patients	No. frames per patient	No. frames
	6	1	6
	26	2	52
	8	4	32
	5	4	20
**Total**	45		110
**Oropharynx**			
	**No. patients**	**No. frames per patient**	**No. frames**
	10	2	20
	8	3	24
	12	4	48
	4	6	24
**Total**	34		116

**Table 2:** Investigated datasets for mucosal SCC segmentation task and corresponding amount of mean percentages of lesion pixels per frame and relative standard deviations.

Dataset	Mean of lesion pixels in %	Standard deviation of lesion pixels in %
Oral cavity	22.84	11.68
Oropharynx	38.04	18.54

Before segmenting the tumor area with FCNNs, the images underwent a cropping procedure to remove black borders. Given the different dimensions and shapes of extracted NBI video-frames, the cropping was customized for each of them. For memory constraints, frames were down-sampled to dimensions of 256×256 pixels to prevent exceeding the available GPU memory (∼14858 MB). Prior to FCNN-based segmentation, images were standardized sample-wise, namely the image mean was removed from each image. Given the small size of the two datasets, data augmentation was performed to avoid overfitting and to increase the ability of the model to better generalize the results. Hence, the training set was augmented by ∼10 times at each cross-validation, imposing the following random transformations to the frames (and corresponding gold-standard masks obtained with manual segmentation): image rotation (random rotation degree in range 0°-90°), shift (random shift in range 0-10% of the frame side length for both width and height), zoom (with zoom values in range 0 and 1), and horizontal and vertical flip.

Three FCNNs were investigated to segment neoplastic images in OC and OP. The architectures tested were:

- U-Net, a fully convolutional U-shaped network architecture for biomedical image segmentation (15);
- U-Net 3, consisting of the previous deep network improved by Liciotti et al. (16) to work with very few training images and yield more precise segmentations;
- ResNet, composed of a sequence of residual units (17).

### Technical Definitions

FCNNs are a type of artificial neural networks that have wide application in visual computing. Their deep hierarchical model roughly mimics the nature of mammalian visual cortex, making FCNNs the most promising architectures for image analysis. FCNNs present an input layer, an output layer, and a variable number of hidden layers, that transform the input image through the convolution with small filters, whose weights and biases are learned during a training procedure.

U-Net is a fully convolutional U-shaped neural networks that is especially suitable for biomedical images. The descending path U-Net is made of repeated 3x3 convolutions and max-pooling, for down-sampling the input image. This path acts as an encoder for feature extraction. The ascending path consists of 3x3 convolutions and up-sampling, for restoring the original input image size. This path acts as decoder for feature processing to achieve the segmentation. The encoder and decoder are linked to each other *via* long skip connections. U-Net3 is inspired by U-Net but introduces batch normalization, which makes the training process faster. ResNet is also divided in two parts: the descending and ascending paths, each consisting of 5 blocks. Each block of the descending path is made of a convolutional sub-block and two identity sub-blocks, whereas in the ascending path there is one up-convolutional sub-block and two identity sub-blocks. The convolutional and identity sub-blocks follow the implementation of (17) and are made of convolution filters. In order to study the complexity of ResNet, in this work we also tested the performance of ResNet considering 1 block, 3 blocks, and 4 blocks per path. In each block, we kept the number of filters for each convolution equal to 16. We also investigated the ResNet with 1 block per path and 8 filters per convolution instead of 16: this represents the simplest model.

### Data Analysis

FCNN performance was evaluated for each network tested and compared to the gold standard represented by manual annotation performed by expert clinicians. A contingency table considering true positive (TP), true negative (TN), false negative (FN), and false positive (FP) results was used. The overall accuracy (Acc) was calculated and defined as the ratio of the correctly segmented area by the algorithm over the annotated area by the expert examiner. The positive and negative samples refer to pixels within and outside the segmented region, respectively. Precision (Prec) was defined as the fraction of relevant instances among the retrieved ones (i.e., positive predictive value = true positives over true and false positives). Recall (Rec) was defined as the fraction of the total amount of relevant instances that were actually retrieved (i.e., true positive rate = true positives over true positives and false negatives). The Dice Similarity Coefficient (Dsc) was evaluated as overlapping measure. The Dsc is a statistical validation metric based on the spatial overlap between two sets of segmentations of the same anatomy. The value of Dsc ranges from 0, indicating no spatial overlap between two sets of binary segmentation results, to 1, indicating complete overlap. Tumor detection performance was evaluated by measuring the computational time required by each of the FCNN architectures investigated to perform automatic segmentation per frame.

Analysis of variance (Anova test) with a significance level of 0.05 was performed to check whether the averages of the computed metrics significantly differed from each other. When significant differences were found, a pairwise T-test for multiple comparisons of independent groups was performed.

## Results

### Oral Cavity Dataset

**Figure 2:**
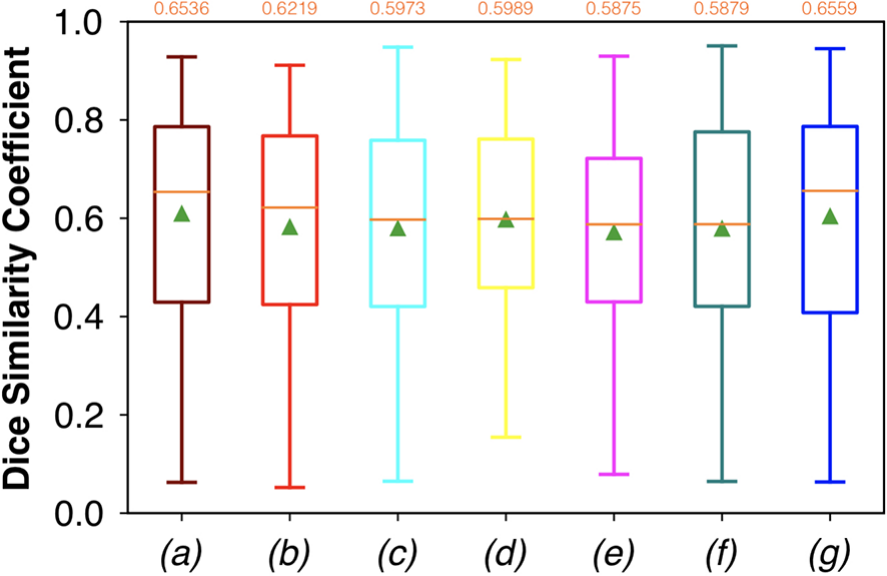
Boxplots of Dsc for the OC dataset, obtained for (a) U-Net architecture, (b) U-Net 3, (c) ResNet with 4(×2) blocks and 16 filters, (d) ResNet with 1(×2) blocks and 8 filters, (e) ResNet with 1(×2) blocks and 16 filters, (f) ResNet with 3(×2) blocks and 16 filters, and (g) ResNet with 5(×2) blocks and 16 filters. Green triangles indicate the mean values, while the orange numbers at the top of each boxplot are the corresponding median values.

**Figure 3:**
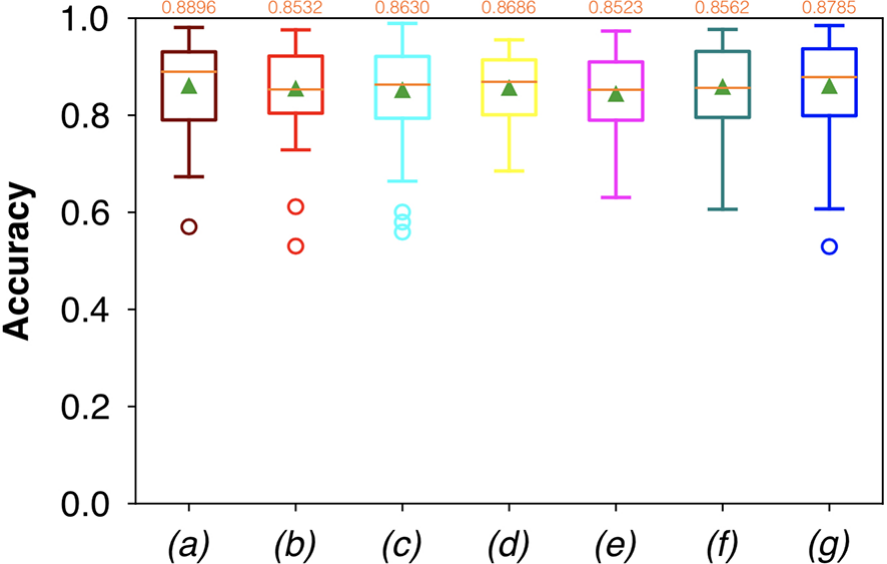
Boxplots of Acc for the OC dataset, obtained for (a) U-Net architecture, (b) U-Net 3, (c) ResNet with 4(×2) blocks and 16 filters, (d) ResNet with 1(×2) blocks and 8 filters, (e) ResNet with 1(×2) blocks and 16 filters, (f) ResNet with 3(×2) blocks and 16 filters, and (g) ResNet with 5(×2) blocks and 16 filters. Green triangles indicate the mean values, while the orange numbers at the top of each boxplot are the corresponding median values.

**Figure 4:**
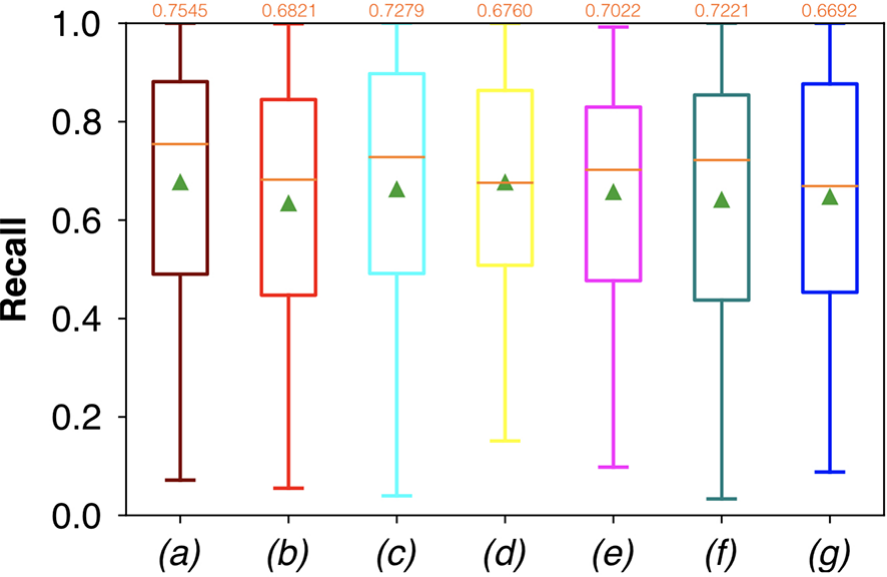
Boxplots of Rec for the OC dataset, obtained for (a) U-Net architecture, (b) U-Net 3, (c) ResNet with 4(×2) blocks and 16 filters, (d) ResNet with 1(×2) blocks and 8 filters, (e) ResNet with 1(×2) blocks and 16 filters, (f) ResNet with 3(×2) blocks and 16 filters, and (g) ResNet with 5(×2) blocks and 16 filters. Green triangles indicate the mean values, while the orange numbers at the top of each boxplot are the corresponding median values.

**Figure 5:**
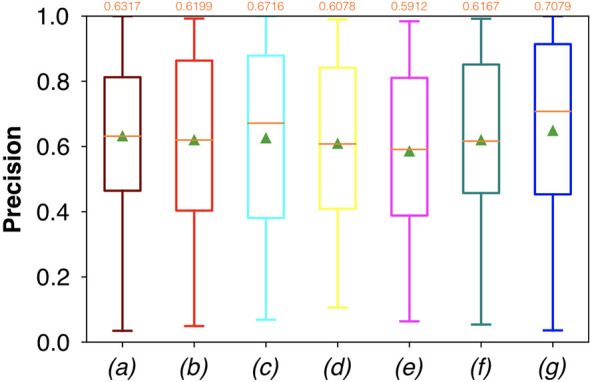
Boxplots of Prec for the OC dataset, obtained for (a) U-Net architecture, (b) U-Net 3, (c) ResNet with 4(×2) blocks and 16 filters, (d) ResNet with 1(×2) blocks and 8 filters, (e) ResNet with 1(×2) blocks and 16 filters, (f) ResNet with 3(×2) blocks and 16 filters, and (g) ResNet with 5(×2) blocks and 16 filters. Green triangles indicate the mean values, while the orange numbers at the top of each boxplot are the corresponding median values.

For FCNN-based segmentation of the OC dataset, the best results in terms of Dsc were achieved by ResNet with 5(×2) blocks (5 for the descending and 5 for the ascending path) and 16 filters, with a median value of 0.6559, as reported in **Figure 2**. The comparison in terms of Acc in **Figure 3** for the tested FCNN architectures showed the best results in terms of median for the U-Net, with a value of 0.8896. Both the abovementioned architectures showed the best results in terms of other metrics. Specifically, ResNet with 5(×2) blocks and 16 filters appeared to be the best in terms of Rec, with a median value of 0.7545, as reported in **Figure 4**. U-Net, in contrast, showed the best result in terms of Prec, with a median value of 0.7079, as reported in **Figure 5**. All FCNNs tested presented very high values of variance, leading to very low values of minima for all metrics evaluated.

No significant difference was found when analyzing variance with the Anova test (p>0.05) to the Dsc, Acc, Rec, and Prec vectors constituted by the metrics of each architecture.

The computational times required by the FCNNs for the automated segmentation task for one image are reported in **Table 3**. It is worth noticing that less deep networks, such as ResNet with 1(×2) blocks and 8 filters, and ResNet with 1(×2) blocks and 16 filters, achieved automated segmentation in shorter times than the others. In particular, ResNet with 1(×2) blocks and 8 filters took only 14 ms to predict a single frame.

**Table 3:** Tested FCNNs and the corresponding times of inference for each single frame, expressed in milliseconds (ms).

FCNNs	Inference time per frame (ms)
**U-Net**	∼115
**U-Net 3**	∼96
**ResNet with 4x2 blocks, 16 filters**	∼66
**ResNet with 1x2 blocks, 8 filters**	∼14
**ResNet with 1x2 blocks, 16 filters**	∼23
**ResNet with 3x2 blocks, 16 filters**	∼59
**ResNet with 5x2 blocks, 16 filters**	∼59

### Oropharyngeal Dataset

**Figure 6:**
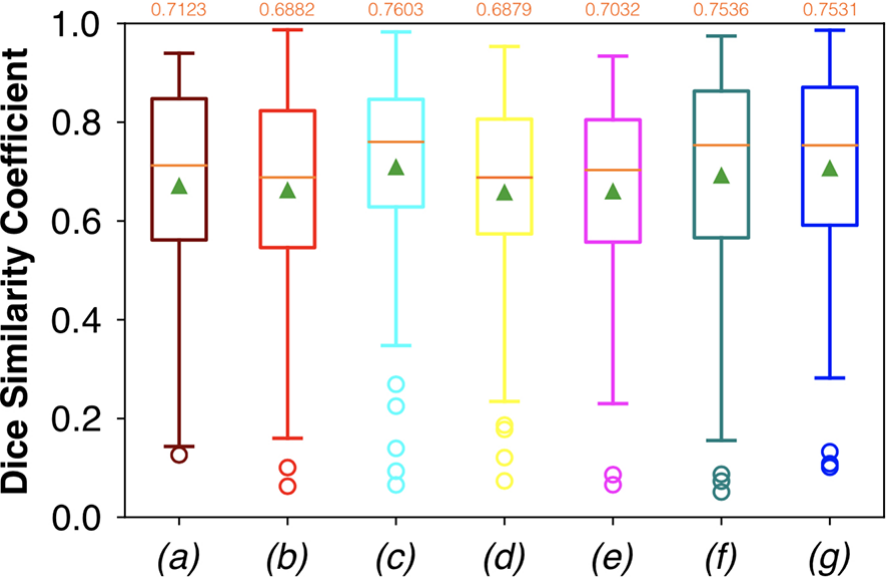
Boxplots of Dsc for the OP dataset, obtained for (a) U-Net architecture, (b) U-Net 3, (c) ResNet with 4(×2) blocks and 16 filters, (d) ResNet with 1(×2) blocks and 8 filters, (e) ResNet with 1(×2) blocks and 16 filters, (f) ResNet with 3(×2) blocks and 16 filters, and (g) ResNet with 5(×2) blocks and 16 filters. Green triangles indicate the mean values, while the orange numbers at the top of each boxplot are the corresponding median values.

**Figure 7:**
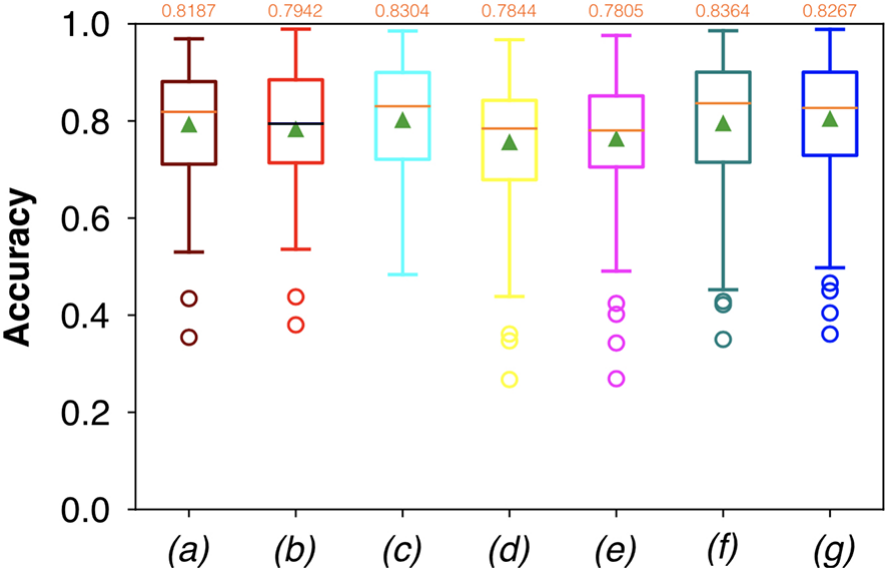
Boxplots of Acc for the OP dataset, obtained for (a) U-Net architecture, (b) U-Net 3, (c) ResNet with 4(×2) blocks and 16 filters, (d) ResNet with 1(×2) blocks and 8 filters, (e) ResNet with 1(×2) blocks and 16 filters, (f) ResNet with 3(×2) blocks and 16 filters, and (g) ResNet with 5(×2) blocks and 16 filters. Green triangles indicate the mean values, while the orange numbers at the top of each boxplot are the corresponding median values.

**Figure 8:**
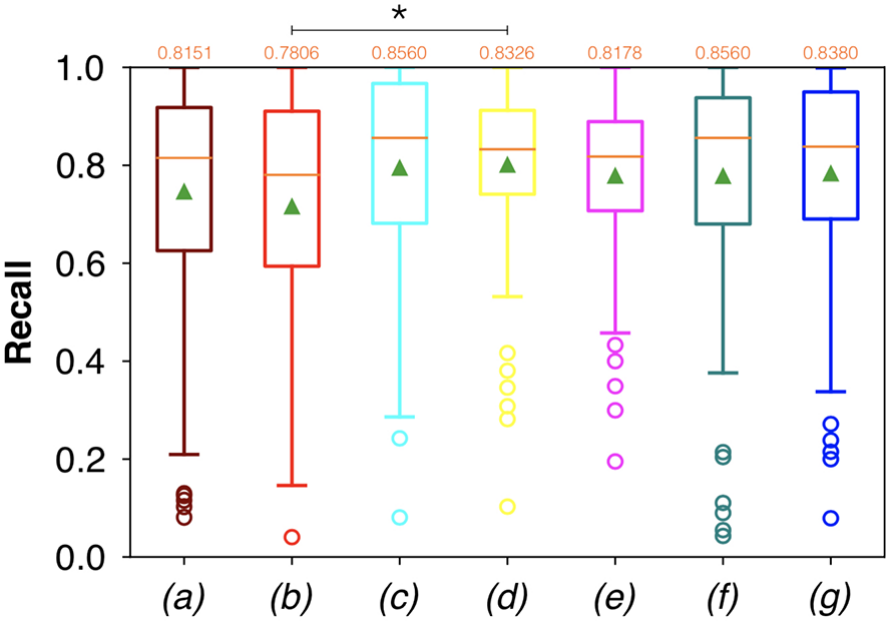
Boxplots of Rec for the OP dataset, obtained for (a) U-Net architecture, (b) U-Net 3, (c) ResNet with 4(×2) blocks and 16 filters, (d) ResNet with 1(×2) blocks and 8 filters, (e) ResNet with 1(×2) blocks and 16 filters, (f) ResNet with 3(×2) blocks and 16 filters, and (g) ResNet with 5(×2) blocks and 16 filters. Green triangles indicate the mean values, while the orange numbers at the top of each boxplot are the corresponding median values. The star indicates a significant difference has been found (Anova test, p<0.05).

Considering FCNN-based segmentation for the OP dataset, the best results in terms of Dsc were achieved by ResNet with 4(×2) blocks and 16 filters, with a median value of 0.7603, as reported in **Figure 6**. The comparison in terms of Acc in **Figure 7** for the FCNN architectures showed the best results in terms of median for the ResNet with 3(×2) blocks and 16 filters, with a median value of 0.8364. Both the abovementioned architectures also showed the best results in terms of Rec, with a median value of 0.8560 for both, as reported in **Figure 8**.

**Figure 9:**
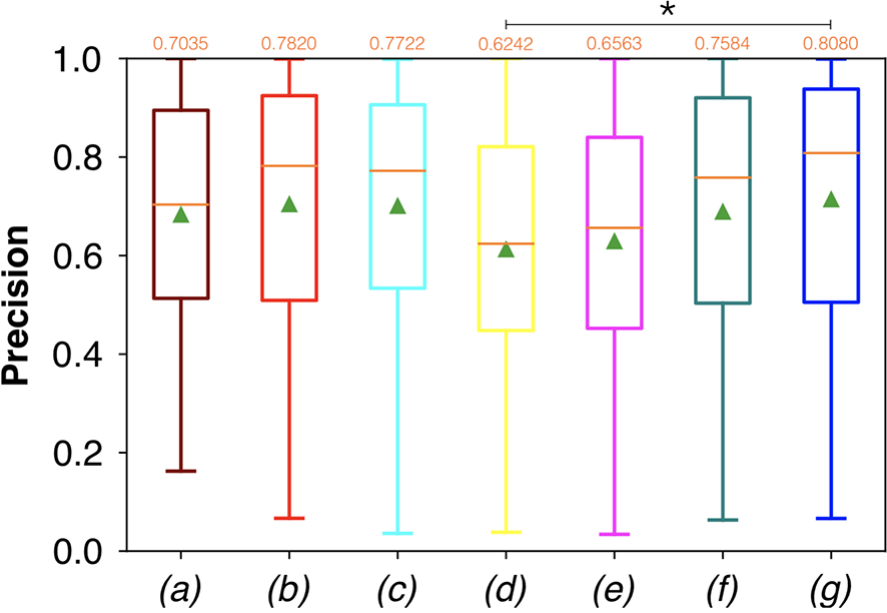
Boxplots of Prec for the OP dataset, obtained for (a) U-Net architecture, (b) U-Net 3, (c) ResNet with 4(×2) blocks and 16 filters, (d) ResNet with 1(×2) blocks and 8 filters, (e) ResNet with 1(×2) blocks and 16 filters, (f) ResNet with 3(×2) blocks and 16 filters, and (g) ResNet with 5(×2) blocks and 16 filters. Green triangles indicate the mean values, while the orange numbers at the top of each boxplot are the corresponding median values. The star indicates a significant difference (Anova test, p<0.05).

Conversely, considering the comparison in terms of Prec in **Figure 9**, the best result was achieved with a deeper network, the ResNet with 5(×2) blocks and 16 filters. However, no significant difference was found when analyzing variance with the Anova test (p>0.05) to the Dsc vectors constituted by the Dsc of each architecture.

A significant difference among the available FCNNs was found applying the same test to the Rec vectors (**Figure 8**). A further investigation was performed using a pairwise T-test for multiple comparisons of independent groups that demonstrated a p value of 0.043 between U-Net 3 and ResNet with 1(×2) blocks and 8 filters, demonstrating that architectures with skip connections (i.e., all the architectures tested except for U-Net and U-Net 3) had greater performances in detecting mucosal sites affected by SCC.

Moreover, a significant difference among the various FCNNs was also found by applying the Anova test to the Prec vectors (**Figure 9**). A further investigation using a pairwise T-test for multiple comparisons of independent groups showed a p value of 0.0454 between ResNet with 5(×2) blocks and 16 filters, and ResNet with 1(×2) blocks and 8 filters, demonstrating that deeper architectures were more precise in detecting SCC.

**Figure 10:**
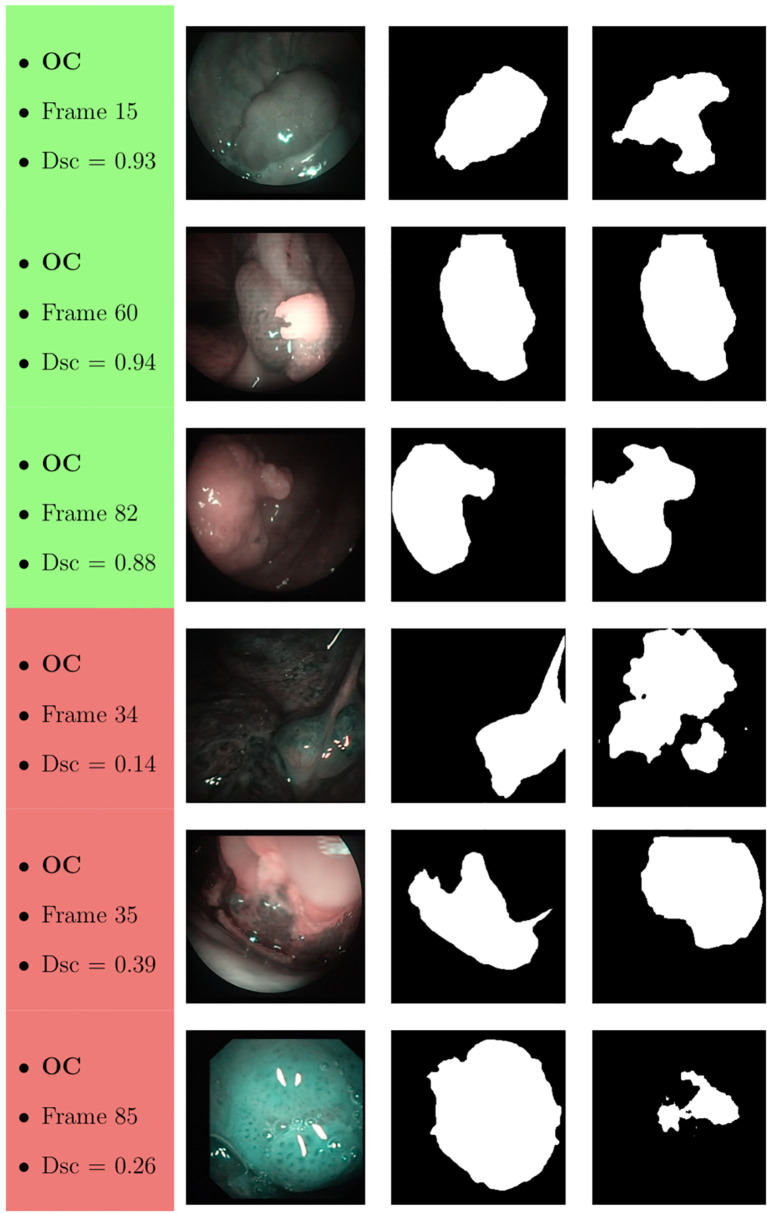
Sample of original OC frames, manual masks, and relative predicted masks for ResNet with 5 (x2) blocks and 16 filters. The red and green boxes correspond to values of Dsc less than 45% (Dsc <0.45) and Dsc greater than 85% (Dsc >0.85), respectively.

Samples of original frames, manual masks, and relative predicted masks for the OC and OP are shown in **Figures 10** **–** **12** in order to provide a visual input on the characteristics of correctly and incorrectly segmented tumors, and non-diagnostic cases.

**Figure 11:**
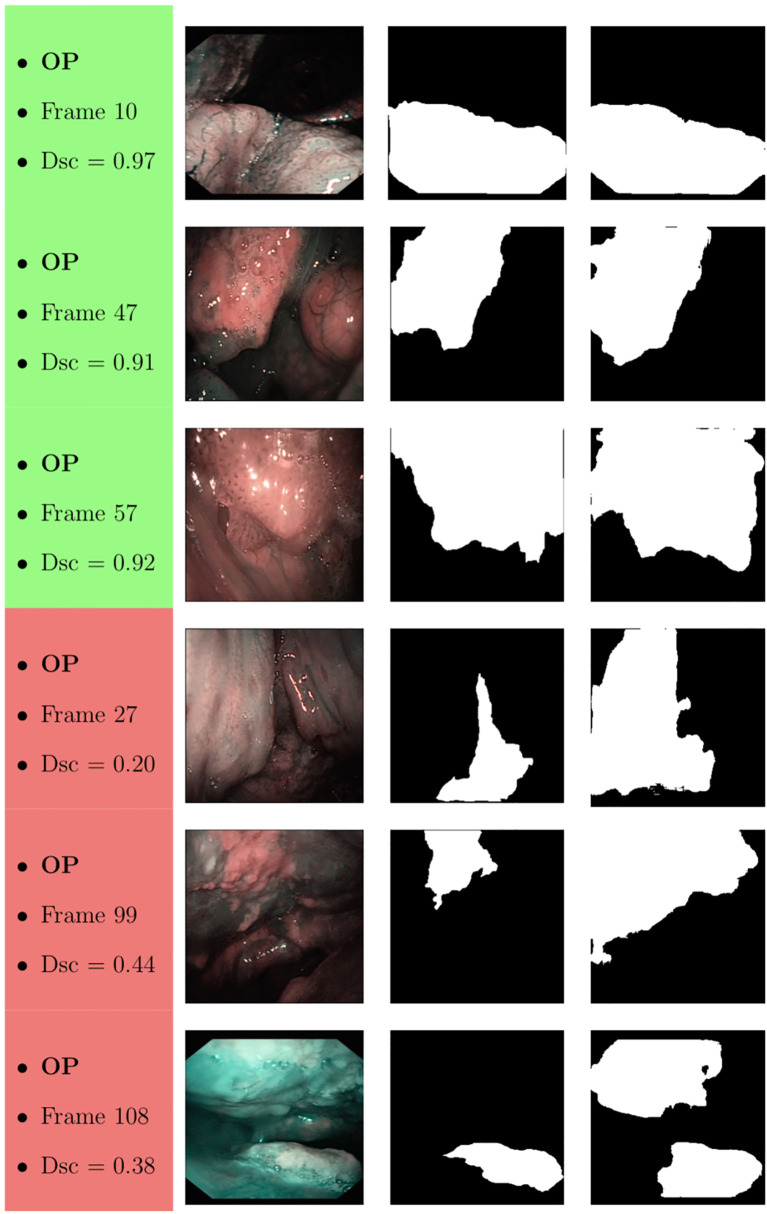
Sample of original OP frames, manual masks, and relative predicted masks for ResNet with 4 (x2) blocks and 16 filters. The red and green boxes correspond to values of Dsc less than 45% (Dsc <0.45) and Dsc greater than 85% (Dsc >0.85), respectively.

**Figure 12:**
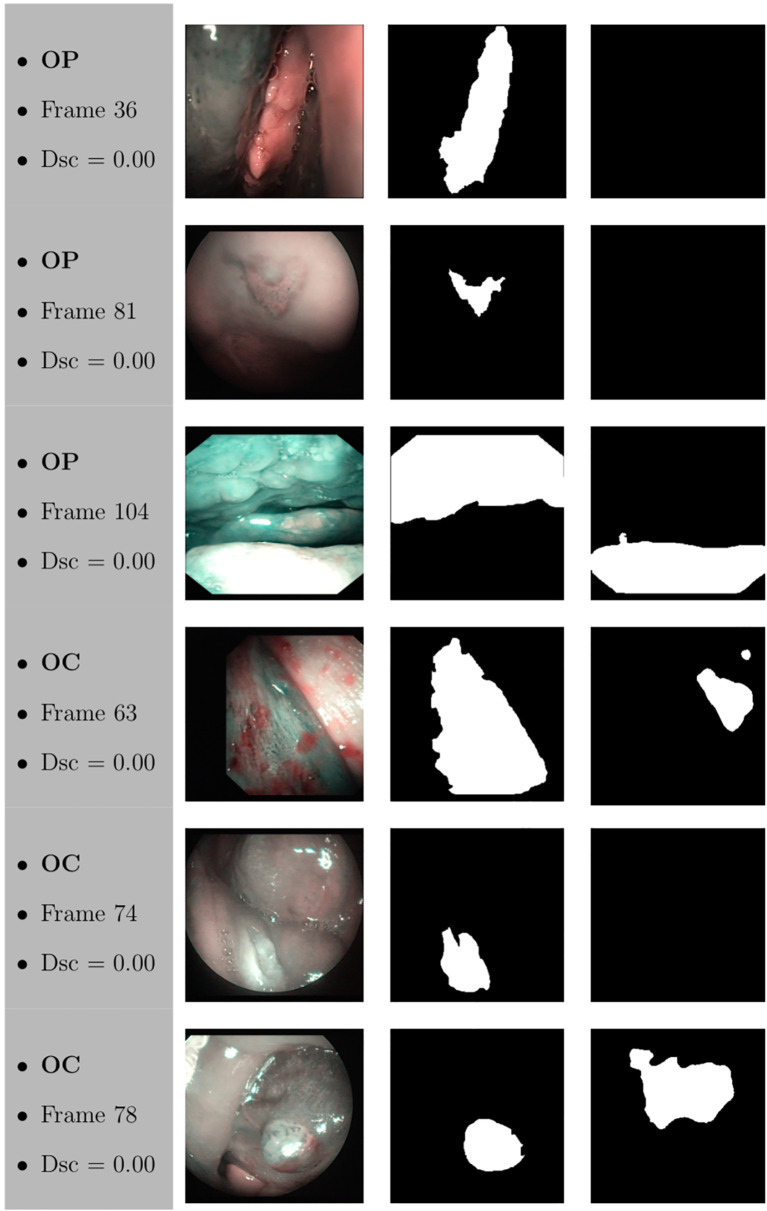
Sample of original frames excluded from the boxplot comparisons due to their Dsc less than 5% (Dsc <0.05) assessed by ResNet with 4 (x2) blocks and 16 filters for OP frames, and ResNet with 5 (x2) blocks and 16 filters for OC frames. The manual masks and relative predicted masks are also reported.

## Discussion

This study presents a computer-aided method for segmentation of SCC through FCNN-based evaluation of NBI video-endoscopic frames afferent to two frequently involved upper aero-digestive tract sites (OC and OP), and evaluates its performance in distinguishing between neoplastic and healthy areas. The overall median Dsc for OC and OP frames of the best performing FCNN [ResNet with 1(×2) blocks and 8 filters] with the shortest time of inference (14 ms) were 0.5989 and 0.6879, respectively.

Of note, this was the first attempt to automatically segment SCC in complex anatomical regions from NBI video-frames. Considering the absence of deep-learning methods in the head and neck literature from which to draw inspiration, this early experience can be considered as a practical approach for segmentation of pathological areas in endoscopic videos, applicable in real time during routine clinical activities, given the short time of inference needed per frame.

Moreover, this approach demonstrates the value of SDS in OC/OP examination and could motivate more structured and regular data storage in the clinic. Indeed, large amounts of data would lead to the possibility of further exploring deep-learning-based algorithms for semantic segmentation, covering a more substantial variability of tissues classification scenarios. In addition, associating such diagnostic videos to subsequently obtained radiologic imaging, pathological specimens, and prognostic characteristics, could pave the way to data mining aimed at understanding adjunctive tumor features (e.g., HPV status, depth of infiltration, risk of regional/distant metastasis) by simple video-endoscopy (18).

In this field, few methodologies have been presented for automatic diagnosis of tumors of the upper aero-digestive tract. As in the present series, most were focused on optimizing the analysis by providing adjunctive features (e.g., NBI, autofluorescence) complementing those obtained by conventional white light endoscopy. Taking advantage of the value of autofluorescence in the OC, Song et al. (19) developed an automatic image classification using a smartphone-based system for OC lesions employing CNNs that evaluated dual-modality images (white light and autofluorescence). The final model reached an accuracy of 87%, sensitivity of 85%, and specificity of 89%.

Conversely, different approaches aimed at maximizing extraction of features focusing on tissue vascularization. Specifically, Barbalata et al. (20) proposed a method for automated laryngeal tumor detection based on post-processing of images. Laryngeal tumors were detected and subsequently classified, focusing on their abnormal intrapapillary capillary loops through anisotropic filtering and matched filter. This further reinforces the rationale of using NBI data for our analysis, since this light-filtering system better highlights blood vessels, thus increasing the quality and quantity of data to be analyzed in each image. This concept was also confirmed by Mascharak et al. (21) who took advantage of naïve Bayesian classifiers trained with low-level image features to automatically detect and quantitatively analyze OP SCC using NBI multispectral imaging. The authors showed a significant increase in diagnostic accuracy using NBI compared to conventional white light video-endoscopy.

Recent studies confirmed the potential of FCNNs in the automatic diagnosis of benign and malignant diseases of the upper aero-digestive tract (22–26), demonstrating an outstanding Acc, comparable with that of experienced physicians. However, these studies were only focused on tumor detection and did not include OC and anterior OP tumors since the examination was only based on transnasal/transoral flexible video-endoscopy. Furthermore, no attempt at segmentation of the precise tumor margins was made.

Considering segmentation and margin recognition tasks, a study by Laves et al. (3) put effort into using FCNN to segment a dataset of the human larynx. The dataset, consisting of 536 manually segmented endoscopic images obtained during transoral laser microsurgery, was tested in order to monitor the morphological changes and autonomously detect pathologies. The Intersection-over-Union metric reached 84.7%. To date, no attempt to a precise visual segmentation by FCNN has been described in the pertinent OC/OP cancer literature.

It should be underlined that our investigation is only a preliminary assessment of feasibility and future potential that could encourage collection of additional evidence and support more extensive studies. The datasets, in fact, were relatively small and partially patient-unbalanced, denoting their high variability. In particular, the OC dataset was composed of a relatively low number of frames in relation to its considerable anatomical complexity and variability of epithelia with different histological and NBI-associated features (27). Moreover, the mean percentage of lesion pixels in each frame was only 22.82 ± 11.68% (with respect to the 38.04 ± 18.54% of the OP dataset). Hence, the high values of Acc might be partially due to the small size of the OC lesions with respect to the entire size of each frame presented in the dataset. Finally, it is worth noticing that all FCNNs tested presented very high values of variance, leading to low values of minima. This is probably due to the difficult task related to the small size of datasets and the significant tissue variability in the regions analyzed. Additionally, OC and OP are characterized by very different endoscopic superficial appearances, with the richness in lymphoid tissue of the latter being one of the most prominent diagnostic obstacles when searching for small tumors of this site even by NBI (27). The lower overall accuracy of FCNNs in the OP observed in the present study may be a sign of such a potential confounding factor.

In general, the type of FCNN did not lead to radical differences in the diagnostic performance in both subsites (while some minor differences may be observed in the OP). The same holds true considering inference times, that were always in the range of “real-time detection” (between 14 and 115 ms). However, in the OP it was possible to observe a higher precision in deeper architectures, demonstrating that an added layer of complexity may improve diagnostic results. Still, deeper architectures were also those needing higher inference times, thus requiring more processing power, and potentially impacting on the aim of real-time segmentation. In this view, when dealing with automatic detection and segmentation of mucosal neoplastic lesions, it will be essential to find a balance between depth of the FCNN and time needed to detect lesions and delineate their margins.

At a subjective evaluation, all FCNNs tended to detect malignant areas where illumination was more prominent, usually in the middle of the picture (**Figures 10** **–** **12**). This factor hints at the importance of optimal and homogeneous illumination, which should be equally distributed throughout the visual field, and not directed only on its central portion. In fact, the operator usually centers the endoscopic image on the lesion to be identified, leading to a significant bias in automatic segmentation by FCNNs. The key role of illumination has also been emphasized by others (22, 23), even showing different diagnostic performances in relation to the types of endoscopic device employed (23). In this view, novel advances in the field of image analysis should be supported by a parallel technical evolution of endoscopes, especially in terms of homogeneous illumination, high definition, colors, and optimization of image clarity.

An adjunctive limitation of this type of studies is that the “ground truth” (i.e., the image segmentation defining true tumor margins) was defined through a single expert opinion. This issue is related to the current impossibility in creating a histopathologic image to be superimposed to the endoscopic view, defining tumor margins at a microscopic level. However, independent evaluations by multiple experts may lead to a more accurate definition of endoscopic tumor margins.

## Conclusions

SDS has promising potential in the analysis and segmentation of OC and OP video-endoscopic images. All tested FCNN architectures demonstrated satisfying outcomes in terms of Dsc, Acc, Rec, and Prec. However, further advances are needed to reach a diagnostic performance useful for clinical applicability. On the other hand, the inference time of the processing networks were particularly short, ranging between 14 and 115 ms, thus showing the possibility for real-time application. Future prospective studies, however, should take into account the number and quality of training images, optimizing these variables through accurate planning and data collection.

## Data Availability Statement

The raw data supporting the conclusions of this article will be made available by the authors, without undue reservation.

## Ethics Statement

The studies involving human participants were reviewed and approved by Ethics Committee of Spedali Civili di Brescia, University of Brescia, Italy. Written informed consent for participation was not required for this study in accordance with the national legislation and the institutional requirements.

## Author Contributions

Study design: AP, SM. Manual segmentation: AP, ST. Revision of segmented images: AP, CP, FB, DL, AD, GP. Analysis: SM, EM, IP, MR. All authors contributed to the article and approved the submitted version.

## Conflict of Interest

The authors declare that the research was conducted in the absence of any commercial or financial relationships that could be construed as a potential conflict of interest.
